# Sinus Disease Grading on Computed Tomography Before and After Modulating Therapy in Adult Patients with Cystic Fibrosis

**DOI:** 10.5334/jbsr.2812

**Published:** 2022-06-14

**Authors:** Corrado Tagliati, Stefano Pantano, Giuseppe Lanni, Davide Battista, Matteo Marcucci, Marco Fogante, Giulio Argalia, Enrico Paci, Gabriella Lucidi Pressanti, Mingliang Ying, Pietro Ripani

**Affiliations:** 1 ASL Teramo, IT; 2 Area Vasta 3, IT; 3 Azienda Ospedaliero Universitaria Ospedali Riuniti, IT; 4 I.N.R.C.A. - IRCCS - POR Ancona - Osimo – Fermo, IT; 5 Department of Radiology, Municipal Central Hospital, Jinhua, Zhejiang, CN

**Keywords:** sinus disease, computed tomography, modulator therapy, CFTR

## Abstract

**Objectives:**

Cystic fibrosis transmembrane conductance regulator (CFTR) modulator therapy effects on respiratory function, pulmonary exacerbations and quality of life have been well documented. However, CFTR modulator therapy effects on sinus disease have not been so well reported. A previous study reported that ivacaftor improves appearance of sinus disease on Computed Tomography (CT) in cystic fibrosis (CF) patients with G551D mutation. The aim of this study was to evaluate the effect of CFTR modulator therapy in sinus disease using CT scores in a wider CF patient population.

**Materials and Methods:**

Forty-eight adult patients with CF underwent at least one CT sinus examination before CFTR modulator therapy (ivacaftor, lumacaftor, ivacaftor/lumacaftor or elexcaftor/tezacaftor/ivacaftor) and one CT sinus examination one year after CFTR modulator therapy initiation. Two radiologists assessed the images in consensus. The Lund-Mackay score (LM score) and the Sheikh-Lind CT sinus disease severity scoring system (SL score) were used. The 22-item SinoNasal Outcome Test (SNOT-22) questionnaire was evaluated before CFTR modulator therapy and one year after CFTR modulator therapy initiation.

**Results:**

CT sinus examination after CFTR modulator therapy showed statistically significant lower mean LM, SL and SNOT-22 scores than CT sinus examination before CFTR modulator therapy (p < 0.001).

**Conclusion:**

Evolution of imaging findings on CT during follow-up closely correlate with improved SNOT-22 score one year after CFTR modulator therapy initiation, indicating that CT may be a useful adjunct during follow-up of CF patients under this treatment as an objective measure of sinonasal disease improvement.

## Introduction

Cystic fibrosis (CF) is a common autosomal recessive disorder, caused by cystic fibrosis transmembrane conductance regulator (CFTR) gene mutations, with highest prevalence in Europe, North America and Australia, affecting about 1 in 2000–3000 Caucasian population newborns [1,2]. CF morbidity and mortality is mainly attributed to lung disease progression and respiratory exacerbations [3]. CFTR protein is not just a chloride channel and its disfunction is at the base of a multisystem disease [4,5]. Among the CF extrapulmonary diseases, chronic rhinosinusitis (CRS) cause significant persistent rhinorrhea, postnasal drip, and thick nasal discharge. Moreover, CRS contribute to sleep disorders which are related to fatigue, reduced concentration and sadness [6,7].

CFTR modulator therapy effects on respiratory function, pulmonary exacerbations, and quality of life have been well documented [8]. However, CFTR modulator therapy effects on sinus disease have not been so well reported. A previous study showed significant improvement with respect to rhinological symptoms and psychological symptoms, using the 20-item Sino-Nasal Outcome Test (SNOT-20) questionnaire [9].

Another previous study reported that ivacaftor improves appearance of sinus disease on Computed Tomography (CT) in CF patients with G551D mutation [10].

The aim of this study was to evaluate the role of CFTR modulator therapy in improving sinus disease using CT scores and a ‘quality of life’ score in a wider CF patient population.

## Material and Methods

### Study population

From March 2017 to February 2022, 148 patients with cystic fibrosis underwent at least one CT sinus examination and were retrospectively collected. Inclusion criteria consisted in a CT sinus examination performed at our hospital before CFTR modulator therapy and a CT sinus examination performed in our institution one year after CFTR modulator therapy (ivacaftor, lumacaftor, ivacaftor/lumacaftor or elexcaftor/tezacaftor/ivacaftor). Pediatric patients (<18-years-old) were excluded. According to these criteria, the study cohort consisted in 48 patients (21 females; 17 males; F:M 1.2:1; mean age 30,2 ± 10.2 years; range 18–67). The study was approved by the institutional review board and ethics committee of our institution. Informed consent was obtained from all individual participants included in the study.

### Computed tomography examination

All CT examinations were performed using a 64 slices CT scanner (Lightspeed VCT 64, GE, Wisconsin, United States) with patients in supine position and head towards the gantry. The scan parameters were as follows: scan range from the hard palate to above the end of the frontal sinuses, 64 × 0.625 mm beam collimation, 100 kVp, 40 mA tube current, 1.0 mm slice thickness reconstructions, coronal and sagittal images multiplanar reconstructions, using both bone and soft tissue reconstruction kernels.

### Computed tomography images evaluation

Two radiologists with at least 10 years’ experience in head CT assessed the images in consensus. CT sinus scoring systems used were the not CF specific modified Lund-Mackay score (LM score) and the more recent CF specific Sheikh-Lind CT sinus disease severity scoring system (SL score) (Tables 1, 2) [11,12]. Both scores need to be evaluated for each side (left and right), and the total score is the sum of the two sides scores. As regard both scores, a higher total score value corresponded to greater severity of the disease. As regard the Lund-Mackay score, only pneumatized sinuses were scored. The raw Lund score was computed for each patient based on the total number of pneumatized sinuses. In addition, to enable the comparison of results, a scaled Lund score was determined by multiplying the raw score by the correction factor 24/(maximum potential Lund score based on number of sinuses pneumatized), thus obtaining a score scaled from 0 to 24 [13].

**Table 1:** Lund-Mackay CT score.


PARANASAL SINUSES	0	1	2

Maxillary	Without abnormalities	Partial opacification	Total opacification

Anterior Ethmoid	Without abnormalities	Partial opacification	Total opacification

Posterior Ethmoid	Without abnormalities	Partial opacification	Total opacification

Sphenoid	Without abnormalities	Partial opacification	Total opacification

Frontal	Without abnormalities	Partial opacification	Total opacification

Ostiomeatal Complex	No obstruction		Obstructed


**Table 2:** Sheikh-Lind CT Sinus Disease Severity Scoring System.


FEATURE	0	1	2	3

Maxillary opacification	None	Mild (<33%)	Moderate (33–66%)	Severe (>66%)

Nasal cavity obstruction	None	Mild (<33%)	Moderate (33–66%)	Severe (>66%)

Displacement of the lateral nasal wall in the middle meatus	None	Yes		

Uncinate process absence/demineralization	None	Yes		

Expansion of any sinuses (mucocele)	None	Yes		


### Sinonasal Quality of Life

The 22-item SinoNasal Outcome Test (SNOT-22) questionnaire evaluated sinonasal quality of life impairment (range: 0–110) [14].

### Statistical analysis

Total scores (LM, SL and SNOT-22 scores) differences before and one year after CFTR modulator therapy were evaluated using Wilcoxon signed-rank test. The statistical significance level was set at p < 0.05. All statistical analyses were performed using MedCalc Software v. 15.8 (Ostend, BEL).

## Results

Mean LM, SL and SNOT-22 scores before CFTR modulator therapy were respectively 11.2 ± 3.3, 8.7 ± 2.4 and 28.6 ± 11.9. Mean LM, SL and SNOT-22 scores one year after CFTR modulator therapy were respectively 8.0 ± 2.7, 6.5 ± 1.8 and 16.2 ± 6.5.

**Figure 1:**
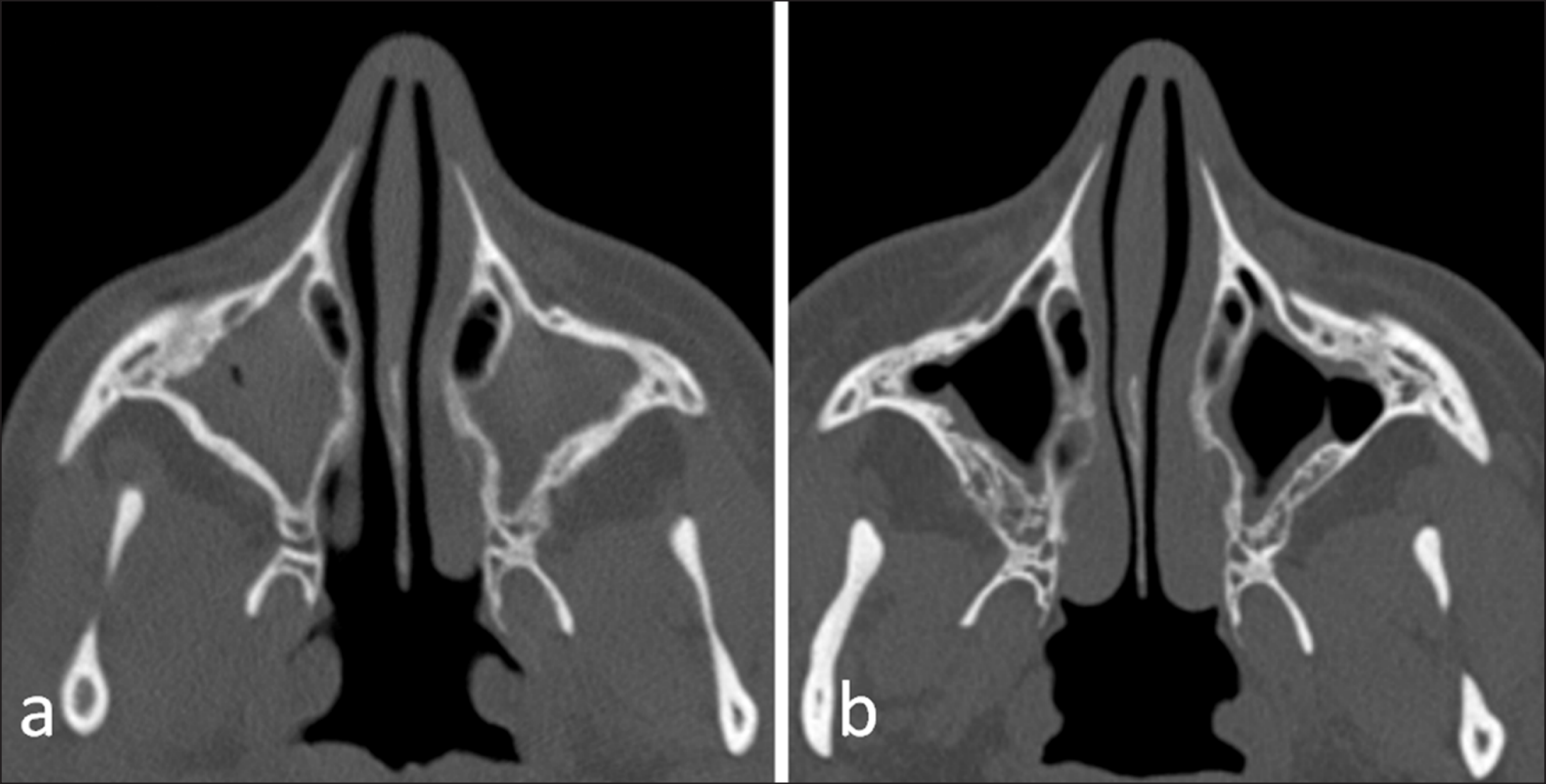
Axial sinus computed tomography images from a cystic fibrosis patient with chronic rhinosinusitis before **(a)** and after **(b)** one year of modulating therapy.

**Figure 2:**
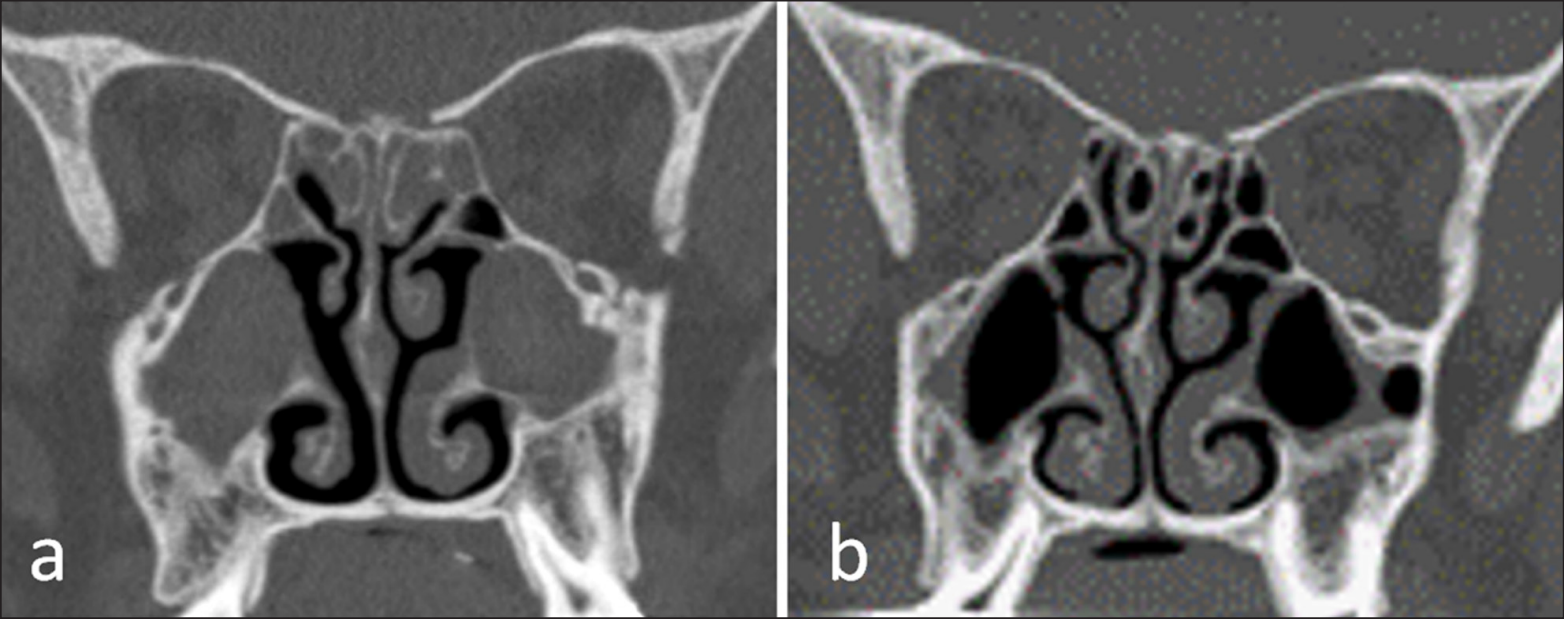
Coronal sinus computed tomography images from a cystic fibrosis patient with chronic rhinosinusitis before **(a)** and after **(b)** one year of modulating therapy.

**Figure 3:**
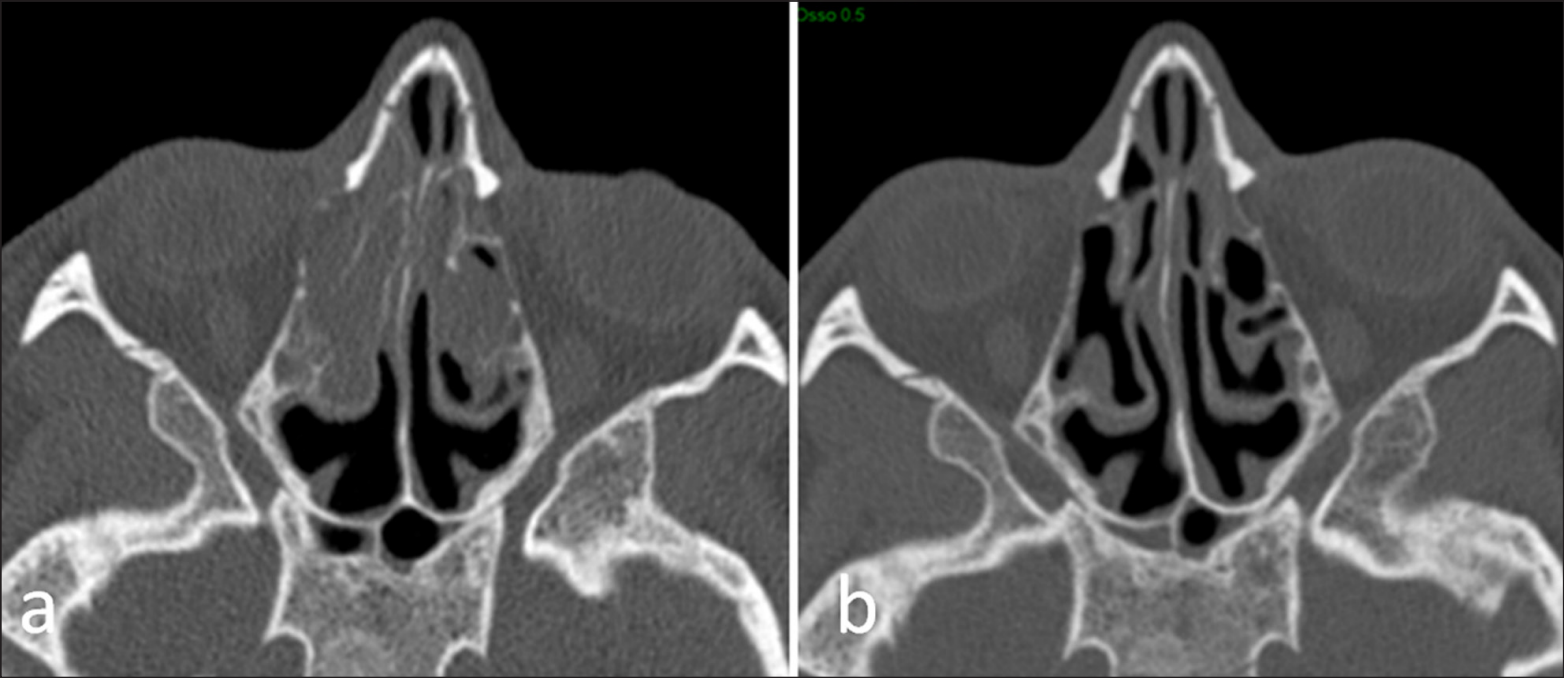
Axial sinus computed tomography images from a cystic fibrosis patient with chronic rhinosinusitis before **(a)** and after **(b)** one year of modulating therapy.

CT sinus examination after CFTR modulator therapy showed statistically significant lower mean LM, SL and SNOT-22 scores than CT sinus examination before CFTR modulator therapy (p < 0.001) (Figures 1, 2 and 3).

## Discussion

Chronic rhinosinusitis is a persistent disease that determines a significant quality of life worsening in CF patients. Therefore, it would be useful to objectively evaluate CFTR modulator therapy effects on sinus disease.

The changes in sinonasal disease severity as visualized on CT and objectified by means of the CT grading scores showed a significant correlation with the improved SNOT-22 score one year after CFTR modulator therapy of adult CF patients in this study.

Improvement in chloride channel function can improve rhinosinusal secretions, change mucus viscosity, reduce inflammation and chronic sinus disease.

A previous study reported that patients with CF and G551D mutation one year after starting ivacaftor therapy showed a significant improvement in sinus disease evaluated by CT [10]. However, in this study the patient population was smaller. Moreover, LM, SL and SNOT-22 scores were not used. Another previous study evaluated eight patients with cystic fibrosis with an S1251N mutation, treated with the potentiator ivacaftor [14]. In this study, CT showed a statistically significant reduction of paranasal sinuses opacification after one year of treatment with ivacaftor. However, in this study the patient population was smaller. Moreover, only LM CT score was used. A recent study about 25 patients with CF showed triple therapy (elexacaftor, tezacaftor and ivacaftor) efficacy on sinus disease. However, in this study with a smaller patient population, the SL score was not assessed. Moreover, a shorter follow-up (six months) after treatment initiation was evaluated [15].

As regards clinical implication of our findings, this study suggests a possible CFTR modulator therapy efficacy in order to reduce rhinosinusal involvement in CF patients. Moreover, as reported by previous studies, CFTR modulator therapy could reduce sinonasal symptoms too, improving quality of life [15,16,17].

The present study has some limitations. First of all, it is a retrospective analysis, with a limited number of patients. Furthermore, patients’ CFTR mutations heterogeneity and the use of different CFTR modulator therapies cannot underline the efficacy of a specific treatment in a specific CFTR mutation. Moreover, the improvement of sinonasal disease one year after CFTR modulator therapy does not prove that the treatment works, as potentially confounding factors that could have contributed to a decrease in sinonasal inflammation in this patient cohort were not actively investigated.

However, to our knowledge, this is the study with the largest CF patient population with sinus disease assessed using two CT scores and SNOT-22 score before and one year after CFTR modulator therapy. Probably, future larger prospective studies that can objectively confirm this suggestion are warranted.

## Conclusion

Evolution of imaging findings on CT during follow-up closely correlate with improved clinical scores (like SNOT-22) during treatment, indicating that CT may be a useful adjunct during follow-up of CF patients under this treatment as an objective measure of sinonasal disease improvement.
